# Vitamin C Pretreatment Enhances the Antibacterial Effect of Cold Atmospheric Plasma

**DOI:** 10.3389/fcimb.2017.00043

**Published:** 2017-02-22

**Authors:** Saga Helgadóttir, Santosh Pandit, Venkata R. S. S. Mokkapati, Fredrik Westerlund, Peter Apell, Ivan Mijakovic

**Affiliations:** ^1^Department of Physics, Chalmers University of TechnologyGöteborg, Sweden; ^2^Systems and Synthetic Biology Division, Department of Biology and Biological Engineering, Chalmers University of TechnologyGothenburg, Sweden; ^3^Chemical Biology, Department of Biology and Biological Engineering, Chalmers University of TechnologyGothenburg, Sweden; ^4^Novo Nordisk Foundation Center for Biosustainability, Technical University of DenmarkLyngby, Denmark

**Keywords:** cold plasma, antibacterial, vitamin C, biofilm, resistance

## Abstract

Bacterial biofilms are three-dimensional structures containing bacterial cells enveloped in a protective polymeric matrix, which renders them highly resistant to antibiotics and the human immune system. Therefore, the capacity to make biofilms is considered as a major virulence factor for pathogenic bacteria. Cold Atmospheric Plasma (CAP) is known to be quite efficient in eradicating planktonic bacteria, but its effectiveness against biofilms has not been thoroughly investigated. The goal of this study was to evaluate the effect of exposure of CAP against mature biofilm for different time intervals and to evaluate the effect of combined treatment with vitamin C. We demonstrate that CAP is not very effective against 48 h mature bacterial biofilms of several common opportunistic pathogens: *Staphylococcus epidermidis, Escherichia coli*, and *Pseudomonas aeruginosa*. However, if bacterial biofilms are pre-treated with vitamin C for 15 min before exposure to CAP, a significantly stronger bactericidal effect can be obtained. Vitamin C pretreatment enhances the bactericidal effect of cold plasma by reducing the viability from 10 to 2% in *E. coli* biofilm, 50 to 11% in *P. aeruginosa*, and 61 to 18% in *S. epidermidis* biofilm. Since it is not feasible to use extended CAP treatments in medical practice, we argue that the pre-treatment of infectious lesions with vitamin C prior to CAP exposure can be a viable route for efficient eradication of bacterial biofilms in many different applications.

## Introduction

In bacterial biofilms, bacteria arrange themselves in three-dimensional structures, which adhere to the surface. Within a biofilm, bacteria interact with each other and an extracellular polymeric matrix provides the community with protection from hostile environment and exposure of antimicrobial agents (Hoiby et al., 2010; Zhou et al., 2015). The extracellular matrix is an important factor in antibiotic resistance. It acts primarily in slowing down the penetration of antibiotics to the cells inside the biofilm, thus reducing the effective dose of antibiotics delivered to the cells. The decreased rate of diffusion of antibiotics into the biofilm enables cellular expression of genes mediating resistance to antibiotics to be activated in the deeper layers of the biofilm (Donlan and Costerton, 2002; Hoiby et al., 2010). Slow penetration of antibiotics inside the matrix material gradually lowers the growth rate of biofilm-associated bacterial cells, which slows down the intake of antibiotics. In addition, the cells killed by the antibiotic in the upper layers of the biofilm “dilute” antibiotics on a per cell basis (Donlan and Costerton, 2002; Bagge et al., 2004; Mai-Prochnow et al., 2008). Biofilms structures also naturally limit the penetration of nutrients to the inner cell layers, and the lack of nutrients results in persistent cells that have higher tolerance to antibiotics (Costerton et al., 1999; Donlan and Costerton, 2002). It has also been proposed that biofilms cannot be completely eradicated due to the survival of persistent cells which accumulate mutations leading to antibiotic resistance (Lewis, 2007). Bacterial cells in biofilms also show increased resistance to the host immune system (Leid et al., 2005; Cerca et al., 2007). Bacterial biofilms are thus recognized as an important cause of chronic infections, including biofilm formation on medical devices, in wounds, and in immunocompromised patients (Costerton et al., 1999; Bjarnsholt et al., 2008). Common bacteria, such as *Staphylococcus epidermidis, Escherichia coli, and Pseudomonas aeruginosa*, that are generally opportunistic or pathogenic, can cause severe chronic infections in immunocompromised individuals (Kaper et al., 2004; de Bentzmann and Plesiat, 2011). The inappropriate use of antibiotics can cause bacteria to develop resistance, rendering infections by antibiotic resistant bacteria which is a major emerging threat (Ventola, 2015a). According to WHO, the global threat of antibiotic resistance is likely to usher a post-antibiotics era (Kostakioti et al., 2013; WHO, 2014). Thus, in addition to managing the usage of antibiotics, there is a significant need of novel approaches for treatment of bacterial infections (Ventola, 2015b). When it comes to bacterial biofilms, antibiotics or other known antimicrobial agents are not strong enough to eradicate them completely (Hoiby et al., 2010; Hoiby, 2011). In this context, the use of a combined therapeutic strategy is recommended to obtain a strong bactericidal effect against bacterial biofilms.

Plasma has been defined as the fourth state of matter, in addition to solid, liquid, and gas (Tendero et al., 2006). The plasma state can be described as an ionized gas, where ionization is obtained by adding energy to the gas. In case of medical applications, the ionizing energy most commonly comes from electricity (Tendero et al., 2006). For plasma to be used in medicine, it has to be generated at atmospheric pressure and the gas temperature must be suitable for treatment of living tissue, with minimal damage to surrounding healthy tissue (Laroussi, 2009). Plasma used in medicine is therefore often called cold atmospheric plasma (CAP; Graves, 2014). In medical treatments, plasma exposure is indirect or remote, where the plasma itself does not come into contact with the tissue, rather, it is an afterglow of the ionized gas (Kong et al., 2009; Bárdos and Baránková, 2010). These plasma sources are called remote plasma jets. In these devices the operating gas flows between two coaxial electrodes, an inner electrode usually powered with radiofrequency (RF) and an outer one grounded. One or both electrodes can be covered with a dielectric barrier (DBD), resulting in an ionized gas jet out of the equipment's nozzle (Bárdos and Baránková, 2010). CAP has received considerable attention in microbiology due to its high bactericidal activity (Maisch et al., 2012; Mai-Prochnow et al., 2014). The main killing mechanism of CAP is the generation of reactive oxygen and nitrogen species (RONS), which have an adverse effect on the cellular biochemistry: damaged proteins and nucleic acids (Joshi et al., 2010; de Geyter and Morent, 2012). In addition, CAP has been shown to have low toxicity toward healthy human cells (Joshi et al., 2010; de Geyter and Morent, 2012). The current CAP-based treatments are not efficient enough in eradicating persistent bacterial biofilms, and we therefore looked for a combined therapeutic strategy which could complement it. Vitamin C, which is a common food additive and can be safely used in medical treatment, is known to be a ROS-generating agent, and can kill some bacterial species (Vilchèze et al., 2013). We investigated if vitamin C pre-treatment could enhance the antibacterial efficacy of CAP at different exposure times on a 48 h bacterial biofilm. The study has been conducted on a non-pathogenic Gram-positive model bacterium *Bacillus subtilis*, and three facultative pathogens: Gram-negative *E. coli* and *P. aeruginosa*, and Gram-positive *S. epidermidis*. The non-pathogenic *B. subtilis* was very sensitive to CAP alone, but for the three pathogens with CAP-resistant biofilms, the pre-treatment with vitamin C dramatically increased the bactericidal effect.

## Materials and methods

### CAP source

For this study we used the CE certificated atmospheric pressure plasma jet kINPen 11 (neoplas control GmbH, Greifswald, Germany). The plasma source consists of an operating device and a hand-held pen. Inside the pen is a ceramic capillary with a centered high-frequency electrode (1 MHz, 2–3 kV). At the end of the pen is a grounded ring electrode that surrounds the capillary. When the working gas flows through the capillary, it gets ionized between the electrodes, creating a plasma-jet expanding out of the nozzle of the hand-held pen. The discharge is generated with a frequency of 5 kHz. The plasma pen was operated with 4.5 standard liters per minute (SLM) of compressed air (2.5 bars).

### Bacterial biofilm cultivation and CAP exposure

A total of 4 bacterial strains were used in this study. *B. subtilis* NCIB 3610 and *E. coli* UTI 89 were grown in LB broth, whereas, *P. aeruginosa* and *S. epidermidis* were grown in tryptic soy broth (TSB). Briefly, overnight grown bacterial culture was diluted to make a 2–5 × 10^6^ CFU/mL suspension in LB broth for *B. subtilis* and *E. coli* and in TSB broth for *P. aeruginosa* and *S. epidermidis*. Two hundred microliters of the diluted bacterial suspension was then loaded on 15 mm glass cover slips and incubated for 24 h at 37°C without agitation. After a 24-h incubation, the old culture medium was replaced by equal volume of fresh medium, and the sample was incubated for another 24 h. The 48 h old biofilms were exposed to CAP for 5, 10, 15, 30, and 60 min (as indicated in the figure legends), with a 15 mm distance between the nozzle and the sample. To evaluate the effect of vitamin C pre-treatment, 48 h biofilms were carefully washed with sterile water to remove the free floating bacteria and treated with 200 μl of 5 mM of vitamin C (for vitamin C and vitamin C + CAP) or same volume of sterile water (for control and CAP) for 15 min followed by a 5-min CAP exposure. Ascorbic acid was purchased from sigma Aldrich (St. Louis, MO, USA).

### Colony forming units (CFU) counting

The viability of bacteria in the biofilms was analyzed by CFU counting. The biofilms were detached from the coverslip by treating with 5 ml of 0.89% NaCl solution, followed by sonication for 10 s (15 s for *S. epidermidis*) to release the bacteria from the glass coverslip. This resulted in a homogenous bacterial suspension in NaCl solution. Homogeneity of the suspension was observed using a brightfield microscope (Supplementary Figure 1). Homogenized suspension (100 μl) was diluted serially and plated on LB agar plates, which were then incubated overnight at 37°C. The number of colonies was then counted and the total number of CFU in 5 ml NaCl was calculated, determining the number of surviving bacteria in the samples.

### Live/dead staining

The biofilms were stained using the Live/Dead BacLight Viability kit L13152, (Invitrogen, Molecular Probes, Inc. Eugene, OR, USA) to determine the proportion of live and dead cells. Bacterial cells with intact cell membranes emit green fluorescence while dead or damaged bacterial cells emit red fluorescence. The staining was performed at room temperature in the dark, for 20 min, using a mixture of 6.0 μM SYTO 9 and 30 μM potassium iodide. Fluorescence microscopic imaging of the biofilms was performed using a Zeiss fluorescence microscope (Axio Imager.Z2m Carl Zeiss, Zena, Germany).

### Scanning electron microscopy (SEM)

For scanning electron microscopy (SEM), the biofilms were fixed with 3% glutaraldehyde solution for 2 h and dehydrated in graded series of ethanol concentrations (30, 40, 50, 60, 70, 80, and 90%) for 15 min in each solution, and finally with 100% ethanol for 20 min. The dehydrated biofilms were dried at room temperature and coated with gold (5 nm) before SEM imaging. SEM imaging was performed with Supra 60 VP (Carl Zeiss AG).

### Statistical analysis

All experiments were performed in biological triplicates and presented as the means ± standard deviations. Intergroup differences were estimated by one-way analysis of variance (ANOVA), followed by a *post-hoc* multiple comparison (Tukey) test to compare the multiple means. Values were considered to be statistically significant when the *P*-value was <0.05.

## Results

### Effect of CAP exposure on the viability of bacterial biofilms

**Figure 1:**
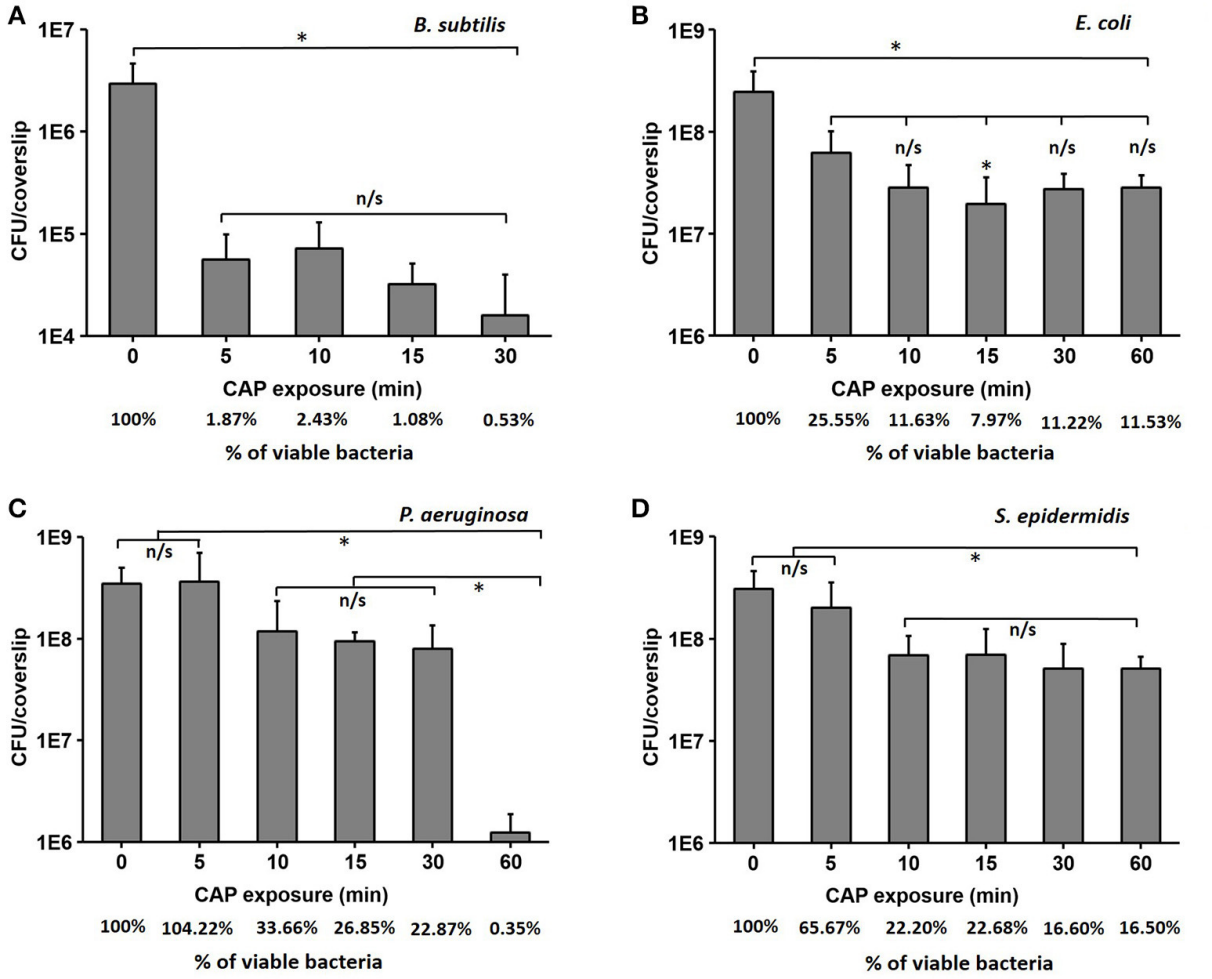
**The loss of viability (log scale) for ***B. subtilis*** (A)**, *E. coli* **(B)**, *P. aeruginosa* **(C)**, and *S. epidermidis* **(D)** after cold atmospheric plasma (CAP) exposure times of 5, 10, 15, 30, and 60 min compared to control samples. The % of viable bacteria was calculated by dividing the total number of bacteria counted in treated sample with total number of bacteria counted in control. All experiments were performed in biological triplicates, the data represent the mean values ± standard deviation. ^*^P < 0.05, n/s is not significant.

Forty-eight hours old biofilms of *B. subtilis, E. coli, P. aeruginosa*, and *S. epidermidis* were prepared as described in materials and methods, exposed to CAP for different time intervals, and the number of surviving cells was estimated by CFU counting. The biofilm of the non-pathogenic *B. subtilis* was clearly the most sensitive, with a strong initial drop of viability by almost two orders of magnitude, followed by a saturated response (final survival after 60 min was 0.5%; Figure 1A). *E. coli* and *S. epidermidis* also showed a significant drop of viability after 5–10 min of treatment, followed by saturation, but their residual resistance to CAP was much higher, with 10–15% of cells surviving after 60 min (Figures 1B,D). Finally, *P. aeruginosa* exhibited a smaller initial decrease in viability, followed by a significant drop after 30 min, leading to a survival of only 0.3% of cells after 60 min of exposure (Figure 1C). The viability of *B. subtilis* was reduced by 2.3 log after 30 min of CAP exposure, which is similar to 5 min of CAP exposure. The viability of *E. coli* was reduced by 0.9 log after 30 min of CAP exposure, which is similar to 5 min of CAP exposure. The viability of *S. epidermidis* was reduced by 0.8 log after 30 and 60 min of exposure. The viability of *P. aeruginosa* was reduced by 0.6 and 2.5 log after 30 and 60 min of CAP exposure respectively (Table 1).

**Table 1:** **The comparison of the log reduction after cold atmospheric plasma (CAP) exposures from 5 to 60 min, a 15-min vitamin C treatment, 15 min di-H_**2**_O + 5 min CAP, and a combined treatment of vitamin C for 15 min followed by a 5-min CAP**.

**Bacterium**	**15-min vitamin C^#^**	**15 min di-H_2_O + 5 min CAP^#^**	**CAP + VC^#^**	**5-min CAP^&^**	**15-min CAP^&^**	**30-min CAP^&^**	**60-min CAP^&^**
*B. subtilis*	1.0	1.9	2.1	2.0	2.0	2.3	x
*S. epidermidis*	0.3	0.2	0.8	0.1	0.6	0.8	0.8
*E. coli*	0.8	1.0	1.7	1.0	1.1	0.9	0.9
*P. aeruginosa*	0.4	0.3	1.5	0.4^α^	0.6	0.6	2.5

*B. subtilis was not treated for 60 min*.

# *Data were obtained in compared to 15 min di-H2O exposed control (from Figure 4)*.

& *Data were obtained in compared to general control (from Figure 1)*.

α *P. aeruginosa was enhanced by 0.4 log*.

**Figure 2:**
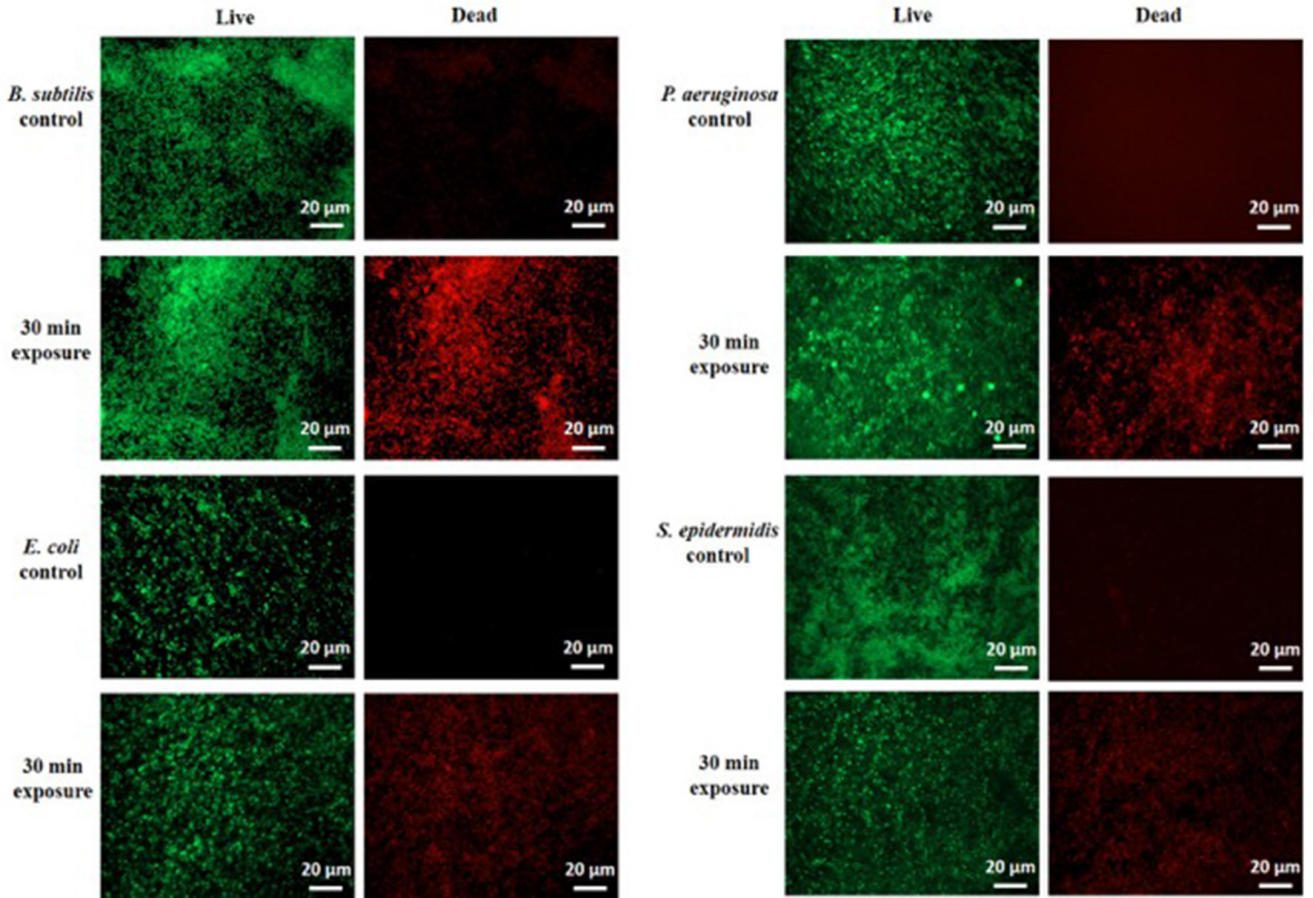
**Live/dead fluorescent staining of ***B. subtilis***, ***E. coli***, ***P. aeruginosa***, and ***S. epidermidis*** control samples and after a 30-min cold atmospheric plasma (CAP) exposure**. Green stained are live cells and red stained are dead cells. All experiments were performed in biological triplicates.

**Figure 3:**
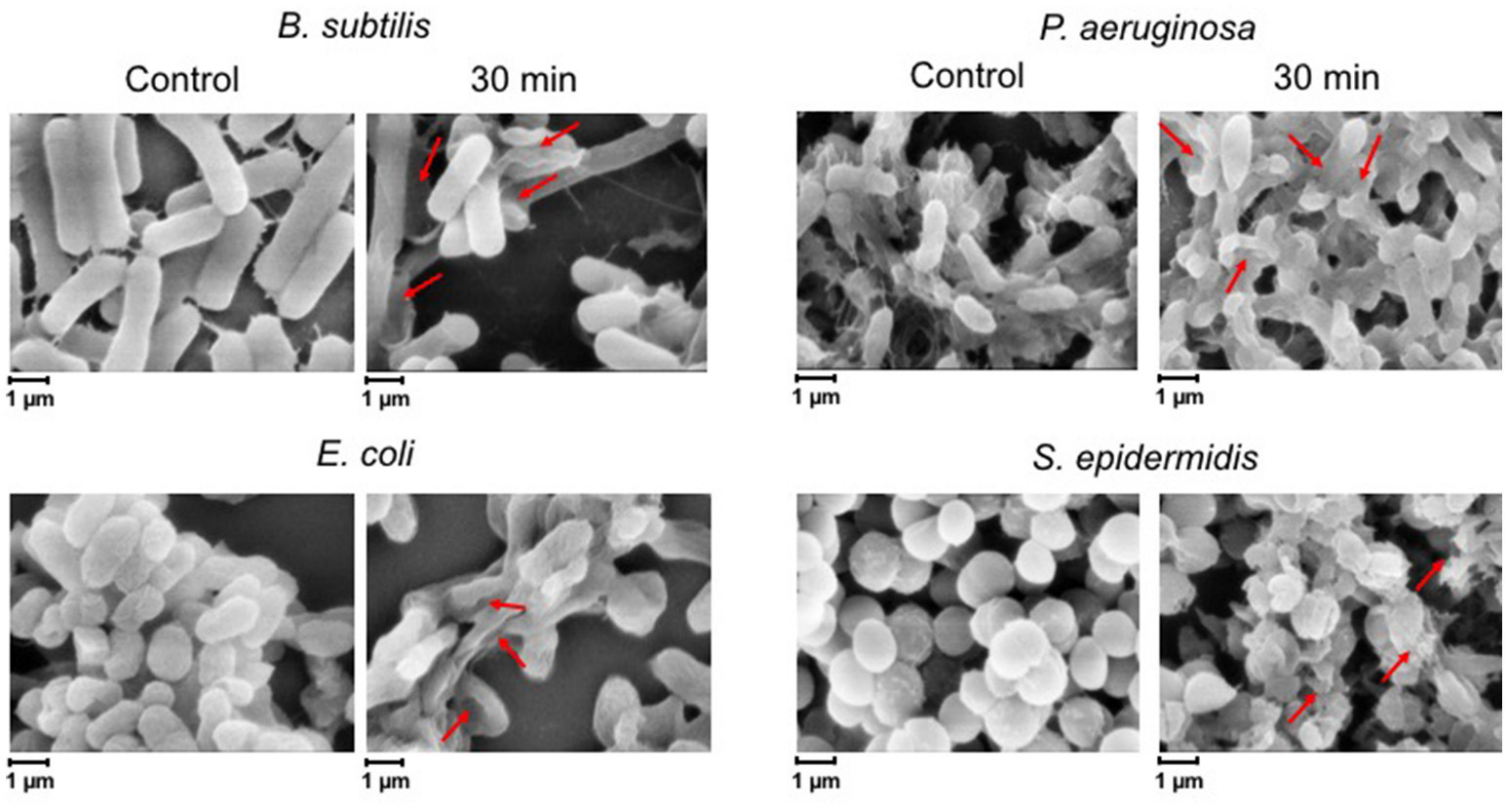
**SEM images of ***B. subtilis***, ***E. coli***, ***P. aeruginosa***, and ***S. epidermidis*** control samples and after a 30-min cold atmospheric plasma (CAP) exposure**. Three individual samples were examined, and the figures show a representative sample. Red arrows indicate the membrane disruption.

To confirm these findings by an independent method, we examined the treated biofilms with live/dead staining using fluorescence microscopy. Figure 2 shows the live/dead fluorescent staining images of all the tested bacterial species after a 30-min CAP exposure. *B. subtilis* biofilm was confirmed as by far the most sensitive to a 30-min exposure. *S. epidermidis, E. coli*, and *P. aeruginosa* biofilms were far more resistant, as expected from the CFU counts. The morphology of the CAP-treated bacteria was examined by SEM. Figure 3 shows SEM images of control samples and samples after a 30-min CAP exposure. Rupture of the bacterial cell membrane can be clearly observed in all treated samples (Figure 3). Pronounced morphological changes were visible in CAP exposed bacterial biofilm. *S. epidermidis* in particular exhibited cell fragmentation and disruption in cell structure typical for ROS-induced stress (Li et al., 2016). Most importantly, biofilms of pathogenic bacteria were not completely annihilated even after a 60 min treatment, which is a very long time in terms of medical treatment.

### Vitamin C pre-treatment strongly enhances the bactericidal effect of CAP

**Figure 4:**
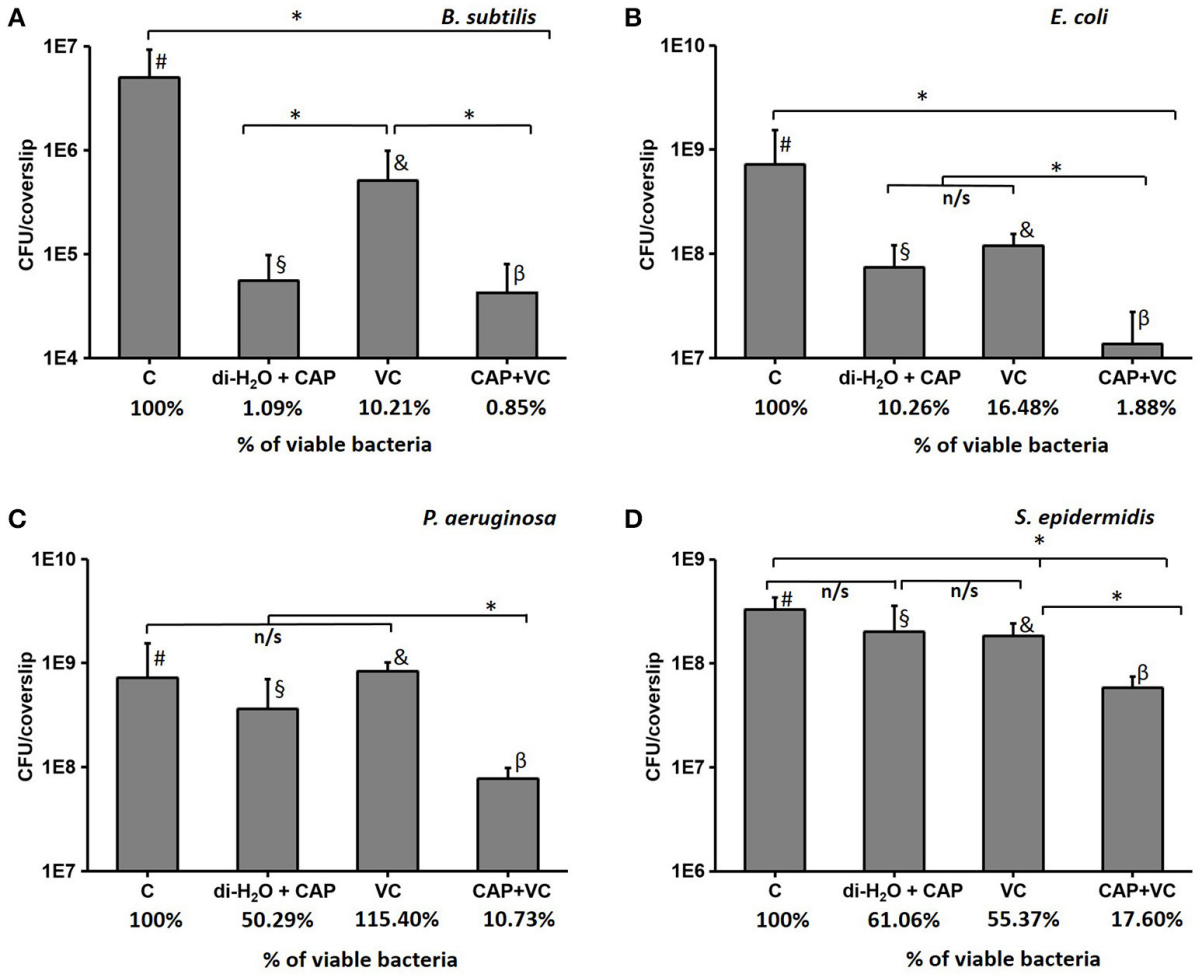
**The number of CFU after a 5-min cold atmospheric plasma (CAP) treatment, a 15-min vitamin C treatment, and the synergistic effect of 15-min vitamin C treatment followed by a 5-min CAP treatment for ***B. subtilis*** (A)**, *E. coli* **(B)**, *P. aeruginosa* **(C)**, and *S. epidermidis* **(D)**. The % of viable bacteria was calculated by dividing the total number of bacteria counted in treated sample with total number of bacteria counted in control. All experiments were performed in biological triplicates, the data represent the mean values ± standard deviation. ^*^P < 0.05, n/s is not significant. #, biofilm treated with di-H2O for 15 min; §, biofilm treated for 15 min with di-H2O + 5 min with CAP; &, biofilm treated with vitamin C for 15 min; β, biofilm treated for 15 min with vitamin C + 5 min with CAP.

**Figure 5:**
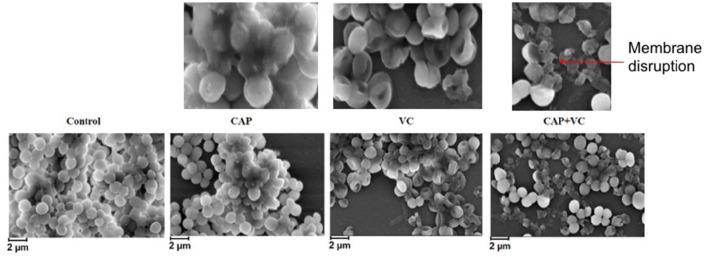
**SEM images of ***S. epidermidis*** samples, after a 5-min cold atmospheric plasma (CAP) exposure, after a 15-min vitamin C treatment, and after a treatment with vitamin C for 15 min followed by a 5-min CAP**. Three individual samples were examined, and the figures show a representative sample.

In order to test our hypothesis that vitamin C may enhance the antibacterial effect of CAP treatment, we pre-treated the biofilm samples with 5 mM ascorbate, for 15 min. After this, the biofilms were treated with CAP for 5 min, which is an acceptably short interval for most medical treatments (Isbary et al., 2010; Wu et al., 2016). Figure 4 shows the comparison of the number of CFUs in control samples that received only the 5-min CAP exposure, the 15-min vitamin C treatment, and both treatments subsequently (vitamin C followed by CAP). Mild decrease in viability of biofilm bacteria was observed with only 5 min of CAP exposure despite the clear synergistic effect was observed with the pretreatment of vitamin C against all pathogen (Figure 4). Vitamin C pretreatment enhances the bactericidal effect of cold plasma by reducing the viability from 10 to 2% in *E. coli* biofilm, 50 to 11% in *P. aeruginosa*, and 61 to 18% in *S. epidermidis* biofilm (Figure 4). Table 1 shows the comparison in log reduction in bacterial viability with vitamin C pretreatment and cold plasma exposure. The pretreatment with vitamin C enhances the log reduction from 0.1 to 0.8 for *S. epidermidis*, 1.0 to 1.7 for *E. coli*, and 0.4 to 1.5 (Table 1). In the case of *B. subtilis*, which is inherently very sensitive to CAP, no significant improvement in bactericidal effect was observed. For all the facultative pathogens, the CAP bactericidal effect was enhanced three- to five-fold by the vitamin C pre-treatment. SEM images of *S. epidermidis* after the individual and combined treatment with vitamin C and CAP are shown in Figure 5. A significantly higher level of structural damage and rupture of the cell membrane was observed in the sample that underwent the combined treatment, compared to only 5 min CAP exposure and 15 min vitamin C treatment (Figure 5, insets).

## Discussion

CAP has emerged as a useful tool for treating local microbial infections and decontamination of various biomedical surfaces (Cahill et al., 2014; Flynn et al., 2015). Previous studies have demonstrated complete eradication of planktonic bacteria after a couple of minutes of CAP treatment (Yu et al., 2006; Flynn et al., 2015). Several studies have also tested the efficacy of cold plasma against the bacterial biofilm (Ermolaeva et al., 2011; Flynn et al., 2015). Their results suggested that bacterial biofilms are more resistant to CAP, and there is a need to increase the duration of exposure for complete decontamination. However, there are no studies regarding the long term cold plasma exposure. Biofilms are considered to be up to 1,000 times more resistant than the planktonic state of bacteria, and it accounts for 60% of human infections, with a high probability of becoming resistant to antimicrobials (Mai-Prochnow et al., 2015). In this study we evaluated the anti-biofilm activity of long term CAP exposure (5–60 min) against four different bacterial strains, one non-pathogenic and three facultative pathogens. Furthermore, we also evaluated whether vitamin C pre-treatment could be used to shorten the duration of the CAP treatment while achieving the same effect.

The response to CAP exposure for both the Gram-negative and Gram-positive bacteria seems to be species dependent. As shown in the Figure 1 and Table 1, non-pathogenic *B. subtilis* is highly sensitive to CAP exposure, dropping to under 2% survival after 5 min. *E. coli* and *S. epidermidis* were remarkably resistant to CAP treatment. These results indicated that biofilms of *E. coli, P. aeruginosa*, and *S. epidermidis* tend to be more resistant to CAP compared to *B. subtilis*, and that different biofilm matrix compositions, which are species-specific, can play a significant role in CAP resistance. The initial viability drop occurred after 5 min of treatment, but then saturation ensued, and the biofilms exhibited as much as 10–15% live bacteria even after 60 min of treatment. *P. aeruginosa* was almost completely eradicated after a 60-min exposure, but its inactivation curve was different. The biofilm survived quite well up to 30 min of treatment, and then the viability dropped radically from 30 to 60 min.

Such “two-slope” inactivation curves were reported previously for biofilm inactivation (Joaquin et al., 2009). The first killing phase corresponds to fast destruction of the top layers of the biofilm, and is followed by the slower destruction of the bottom layers, which are covered by dead cells and exopolysaccharide biofilm matrix. It was also reported that the CAP treatment triggers the bacterial cell entry into a viable-but-non-cultivable (VBNC) survival state during the initial drop in the inactivation curve, followed by the second phase in which the cells are actually killed. The VBNC state has been reported for many Gram-negative bacteria, which undergo morphological changes and decrease in size (Joaquin et al., 2009).

Since the CFU method does not differentiate the dead cells from the ones in the VBNC state, it is important to combine it with other methods for confirmation. Our live/dead fluorescent staining images (Figure 2) confirm the 30-min results of the CFU counting, with *B. subtilis* being most sensitive, and the three facultative pathogens comparably more resistant. Taken together, the CFU counts and live/dead staining suggest that a large portion of *P. aeruginosa* cells might be in the VBNC state up to 30 min of exposure, and are finally killed after 60 min.

For all the tested facultative pathogens, a 5 min CAP treatment (which is a feasible duration for medical treatments) did not efficiently eliminate the biofilm. The survival after 5 min of CAP treatment was 25% for *E. coli*, 65% for *S. epidermidis*, and 100% for *P. aeruginosa* (Figure 1). Since the bactericidal effect of CAP is based on oxidative stress due to generation of ROS, we investigated whether the efficiency of CAP could be enhanced by pre-treatment with other ROS-generating agents known to be harmless to humans. Vitamin C, an important dietary supplement for humans, also has been demonstrated to enhance the antimicrobial activity of several antimicrobial agents (Cursino et al., 2005; Khameneh et al., 2016). Therefore, we pre-treated the biofilms for 15 min with 5 mM vitamin C, also known to generate ROS in most bacterial cells (Vilchèze et al., 2013). The result was very encouraging. Vitamin C alone lead to some loss of viability, but most importantly, when followed by a 5 min CAP treatment, the viability of biofilms was reduced to only 1.9% for *E. coli*, 17.6% for *S. epidermidis*, and 10.7% for *P. aeruginosa* (Figure 4). This was a dramatic improvement (three- to five-fold) over the 5 min CAP treatment alone. Our results indicate that in combination with vitamin C, a very short CAP exposure can be efficient against resilient biofilms of bacterial pathogens. The powerful synergistic effect of CAP combined with vitamin C is clearly visible in the Figure 5, where the treatment is affecting the membrane integrity. Vitamin C treated cells were more susceptible to collapsing during sample processing and as a result more altered *S. epidermidis* cells were observed followed by complete disintegration of cellular structure after subsequent exposure to cold plasma. The pretreatment with vitamin C might show great impact on current CAP based treatment strategy such as wound and root canal treatment by enhancing the bactericidal effect. *In vivo* studies are needed to evaluate the effect of proposed combined treatment on real multispecies biofilm consortium.

Overall, our results suggest that a brief pre-treatment with vitamin C, followed by a very short CAP treatment could be a viable route for treating various types of infectious lesions.

## Author contributions

SP, SH, and VM designed and carried out the experiments. SP, SH, VM, and IM analyzed the data and wrote the manuscript. FW and PA made substantial contributions to conception and design of the work and analysis and interpretation of data, and they critically revised the manuscript.

## Funding

This work was funded by grants from the Chalmers University of Technology and VINNOVA to IM and FW, and ÅForsk to IM.

### Conflict of interest statement

The authors declare that the research was conducted in the absence of any commercial or financial relationships that could be construed as a potential conflict of interest.
