# Supplementary figures and images for: Vitamin C Pretreatment Enhances the Antibacterial Effect of Cold Atmospheric Plasma
Source: Front Cell Infect Microbiol. 2017 Feb 22;7:43. doi: 10.3389/fcimb.2017.00043 (PMC5319976; doi:10.3389/fcimb.2017.00043)

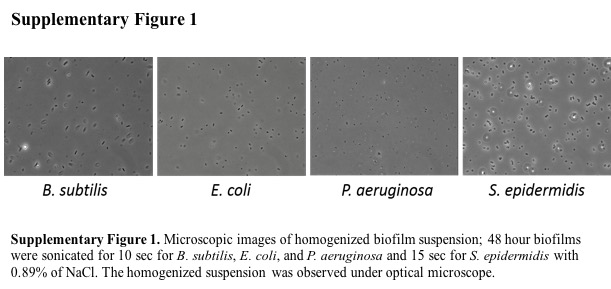

Supplement: Supplementary file 1 [file Image1.jpeg]
